# The Role of Dietary Energy and Macronutrients Intake in Prevalence of Irritable Bowel Syndromes

**DOI:** 10.1155/2019/8967306

**Published:** 2019-05-16

**Authors:** Jing-jing Zhang, Han Ma, Jin-zhou Zhu, Chao Lu, Chao-hui Yu, You-ming Li

**Affiliations:** ^1^Department of Gastroenterology, The First Affiliated Hospital, College of Medicine, Zhejiang University, Hangzhou 310003, China; ^2^Department of Gastroenterology, The First Affiliated Hospital of Soochow University, Suzhou 215006, China

## Abstract

**Background:**

Irritable bowel syndrome (IBS) is a chronic gastrointestinal disorder characterized by abdominal pain and altered bowel habits in the absence of any detectable organic illnesses. Interest in the effect of dietary opponents to the IBS pathogenesis has been increased in recent years. This study aims to review previous studies to determine the relationship between IBS prevalence in community and dietary energy and macronutrients intakes according to the national nutrition surveys.

**Methods:**

A literature search was conducted in PubMed and EMBASE to September, 2018, to identify population-based studies that reported the prevalence of IBS. Daily energy intake, daily carbohydrates, and protein and fat percent contribution to energy intake (%) were obtained from study population-based national nutrition survey. The correlations of prevalence of IBS and dietary intakes were obtained by Spearman coefficient or Pearson coefficient.

**Results:**

Global prevalence of IBS was 11.7%. There was no correlation between overall prevalence of IBS of individual countries and national energy intake (P = 0.785), protein proportion (P = 0.063), carbohydrates proportion (P = 0.505), or fat proportion (P = 0.384) according to the years when the studies were conducted. No correlations were detected between dietary intake and male or female IBS prevalence. Interestingly, protein proportion was positively correlated with the prevalence of IBS in Rome III criteria (r = 0.569).

**Conclusion:**

Our findings demonstrate that dietary energy and macronutrients intake do not play a direct role in prevalence of IBS. However, IBS diagnostic criteria seem to have a bias on the correlation between prevalence of IBS and dietary intake. Further studies are needed to confirm the correlation between prevalence of IBS and specific dietary intake.

## 1. Introduction

Irritable bowel syndrome (IBS) is a chronic gastrointestinal disorder characterized by abdominal pain and altered bowel habits in the absence of any detectable organic illnesses [1]. The prevalence of IBS varies from 1 to 30% in the community, with a pooled global prevalence of 11.2%. In most recent Rome IV diagnostic criteria, IBS seems to affect 5-12% of the population worldwide. IBS develops more frequently in women compared with men and is more commonly diagnosed in patients aged between 30 and 50 years old [2].

The natural history of IBS is also distinguished by relapsing and remitting symptoms, like Crohn's disease and ulcerative colitis, which means that it would lay a considerable economic burden on the country. And despite of its functional nature, IBS patients still exhibit similar degree of impairment of life quality as other chronic diseases such as diabetes, hypertension, and inflammatory bowel diseases [3, 4].

The pathophysiology of IBS still remains uncertain, but multiple factors appear to contribute to its pathogenesis, including gastrointestinal motility disturbance, visceral hypersensitivity, intestinal inflammation, postinfection, altered fecal microflora, small intestinal bacterial overgrowth, food sensitivity, genetic susceptibility, and psychosocial dysfunction. Current therapy for IBS is limited and almost based on symptoms, including psychological interventions, dietary manipulation, and pharmacologic agents [5–8].

Interest in the effect of dietary opponents to the IBS pathogenesis has been increased in recent years. Many IBS patients report problems with some specific foods, such as milk and milk products, wheat products, caffeine, certain meat, cabbage, onion, peas/beans, hot spices, fried food, smoked products, and alcoholic beverages [9, 10]. However, the importance of dietary factors in IBS is controversial. Detailed studies of the relationships between diet and symptoms in IBS are limited [11–14]. This study aims to review previous studies to determine the relationship between IBS prevalence in community and dietary energy and macronutrients intakes according to the national nutrition surveys.

## 2. Methods

### 2.1. Search Strategy and Study Selection

A literature search was conducted by a MEDLINE search in PubMed and EMBASE to September, 2018, using the string ‘((irritable bowel syndrome) OR (IBS) OR (spastic colon) OR (irritable colon) OR (functional adj5 bowel) OR (mucous colitis) OR (mucous colitides)) AND (prevalence OR incidence OR epidemiology)' and was limited to humans. A recursive search was performed by using the bibliographies of all obtained articles. Studies included were limited to cross-sectional surveys fully published that reported the prevalence of IBS and recruited subjects from general population or community. Those who reported prevalence in convenience samples, such as university students, employees in a certain company, people attending clinic health screening, and outpatients or inpatients in certain hospitals, were not eligible for inclusion. Also, studies had to recruit at least 50 adults (15 years old and older). The definition of IBS required one or more of the following criteria: the Manning criterion, Rome I, Rome II, and Rome III criteria, or a specific questionnaire. There were no language restrictions. Foreign language articles were translated if needed. Eligibility evaluation was performed by two investigators independently. Any disagreements on study eligibility were resolved by consensus.

### 2.2. Data Extraction

Data were extracted independently by two investigators to a Microsoft Excel spreadsheet (2013 edition; Microsoft, Redmond, WA), again with any discrepancies resolved by consensus. The following data were collected for each study: first author, publish year, study year, country or region, criteria used to define IBS, number of subjects, number of subjects with IBS, number of female or male subjects, number of female or male subjects with IBS, age distribution information of IBS, and percentage of each subtype of IBS according to predominant stool pattern (constipation-predominant IBS [IBS-C], diarrhea-predominant IBS [IBS-D], mixed stool pattern IBS [IBS-M], and unclassifiable IBS [IBS-U]). On condition that IBS prevalence was provided according to more than one diagnostic criterion in an individual study, the data depending on each criterion were extracted as independent data.

### 2.3. Estimation of National Dietary Intake

We extracted daily energy intake (kcal/day), daily carbohydrates, and protein and fat percent contribution to energy intake (%) from study population-based national nutrition survey. And the conducted time of the selected national nutrition survey must be closest to the study period of that article. When the national nutrition survey was not available, we applied population or community-based dietary data published for replacement. On condition that an article failed to offer precise time and duration that studies were undertaken, the time was estimated according to the following equation: study year = publish year - mean gap between attainable publish and study year (4.62 years, based on the available data).

### 2.4. Data Synthesis and Statistical Analysis

In this study, the influence of dietary intake on the prevalence of IBS was estimated. Meanwhile, IBS prevalence was analyzed according to geographic location, diagnostic criteria, study year, and gender. Moreover, composition of IBS subtypes and age distribution of subjects with IBS were analyzed. The correlations of prevalence of IBS and dietary intakes were obtained by Spearman coefficient or Pearson coefficient. If the variables satisfied normal distribution, we chose Pearson correlation, or otherwise Spearman correlation. The linear or weighted least square (WLS) regression analysis was applied followed by a significance in Pearson or Spearman correlation analysis. Residuals were analyzed using the Durbin–Watson test, in which d_U_ < DW < (4-d_U_) was defined as no significant residual autocorrelation. Comparisons between two groups were conducted using Student's* t*-test, and multiple-group analyses were conducted by the one-way ANOVA test, followed by the Tukey-Kramer post hoc test. P values less than 0.05 were considered significant. Statistical analysis was performed using SPSS 20.0 (IBM, Chicago, IL, USA). Associated data were calculated and plotted by Prism 5 (Graph Pad, San Diego, CA, USA).

## 3. Results

### 3.1. Process of Articles Evaluation

**Figure 1:**
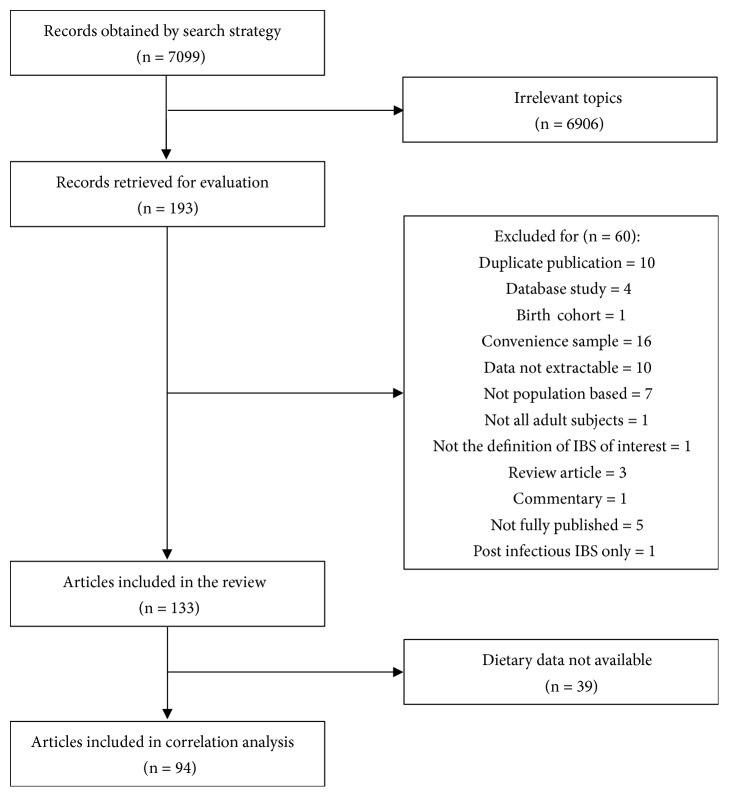
Flow diagram of assessment of studies identified in the systematic review and meta-analysis.

It was presented in Figure 1 that 7099 records were obtained by the search strategy and 193 articles that appeared to be relevant to the topic were included for the further evaluation. Finally, there were 133 articles that fulfilled the eligibility criteria and represented 163 individual data (Table S1 in the Supplementary Materials). Among the 133 eligible articles, 39 articles could not find corresponding national dietary data (45), leaving 94 articles (118 individual data) for the analysis of correlations between prevalence of IBS and dietary intake.

### 3.2. Global Prevalence and Characteristics of IBS

**Figure 2:**
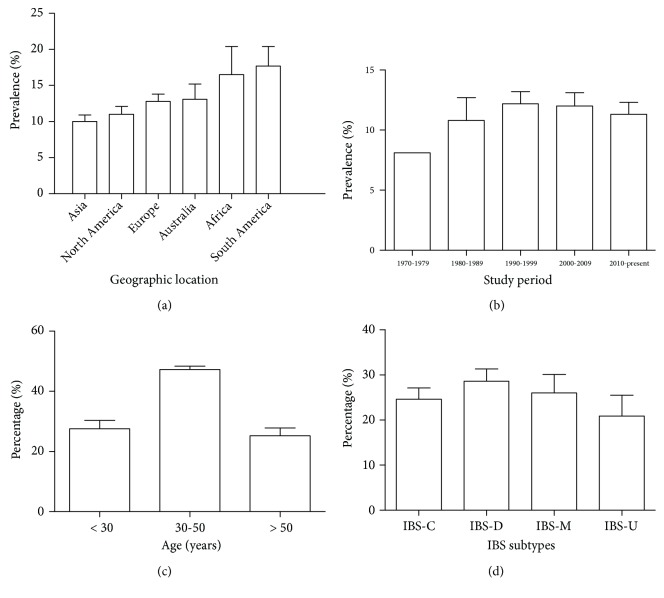
Global prevalence of irritable bowel syndrome (IBS) (data were expressed as mean ± standard error). (a) The prevalence of IBS in Asia, North America, Europe, Australia, Africa, and South America. (b) The prevalence of IBS in 1970-1979, 1980-1989, 1990-1999, 2000-2009, and 2010-present. (c) The age distribution of subjects with IBS. (d) The predominant stool pattern in IBS patients (constipation-predominant IBS [IBS-C], diarrhea-predominant [IBS-D], mixed stool pattern [IBS-M], and unclassifiable [IBS-U]).

The majority of studies were conducted in North America (25/163), Europe (53/163), and Asia (65/163). There were few studies from Africa (5/163), South America (6/163), and Australia (9/163). Global prevalence of IBS was 11.7% (95% confidence interval [CI], 10.6-12.9%). The prevalence of IBS according to geographic location was 10.0% (95% CI, 8.2–11.9%), 11.0% (95% CI, 8.7–13.3%), 12.8% (95% CI, 10.8–14.9%), 13.1% (95% CI, 8.2–18.0%), 16.5% (95% CI, 9.4–23.5%), and 17.7% (95% CI, 6.9–28.5%) in Asia, North America, Europe, Australia, Africa, and South America, respectively (Figure 2(a)). There was a significant difference between the geographic prevalence of IBS (F = 2.324, P = 0.045, ANOVA); however, when followed by the Tukey-Kramer post hoc test, the significance disappeared.

Most studies were conducted in 1990-1999 (45/163), 2000-2009 (66/163), and 2010-present (40/163). Only few studies were carried out in 1970-1979 (2/163) and 1980-1989 (10/163). The prevalence of IBS according to study year was 8.1% (95% CI, 8.1–8.1%), 10.8% (95% CI, 6.6–15.0%), 12.2% (95% CI, 10.1–14.2%), 12.0% (95% CI, 9.9–14.1%), 11.3% (95% CI, 9.3–13.2%), and 17.7% (95% CI, 6.9–28.5%) from the earliest period to the latest period (Figure 2(b)). No significant difference was detected between these groups (F = 0.252, P = 0.908, ANOVA).

There were 39 studies providing data about age distribution of subjects with IBS, in which only 14 studies used identical age bands. About half of IBS patients are 30–50 years old (47.3% [95% CI, 45.0–49.3%]), and those who are younger than 30 years and older than 50 years were 27.5% (95% CI, 22.3–34.0%) and 25.2% (95% CI, 19.4–29.6%), respectively (Figure 2(c)).

43 studies reported the predominant stool pattern in those with IBS and 26 studies reported the data based on the IBS-C, IBS-D, IBS-M, and IBS-U subtypes. The proportions of IBS-C, IBS-D, IBS-M, and IBS-U were 24.6% (95% CI, 19.9–29.9%), 28.6% (95% CI, 23.3–34.1%), 26.0% (95% CI, 18.0–33.8%), and 20.9% (95% CI, 12.6–30.7%) worldwide, respectively (Figure 2(d)).

### 3.3. Prevalence of IBS with Dietary Intake

**Figure 3:**
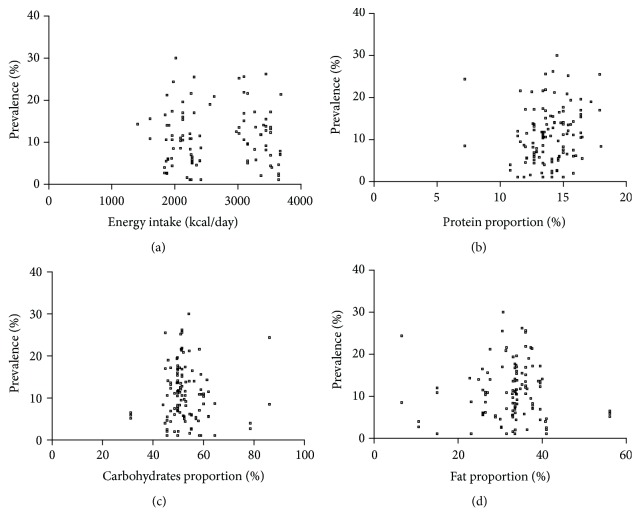
The scatterplot of prevalence of irritable bowel syndrome (IBS) and dietary factors. (a) The scatterplot of prevalence of IBS and energy intake (r = -0.027, P = 0.785, Spearman correlation). (b) The scatterplot of prevalence of IBS and protein proportion (r = 0.172, P = 0.063, Pearson correlation). (c) The scatterplot of prevalence of IBS and carbohydrates proportion (r = -0.062, P = 0.505, Spearman correlation). (d) The scatterplot of prevalence of IBS and fat proportion (r = 0.081, P = 0.384, Spearman correlation).

There was no correlation between overall prevalence of IBS of individual countries and national energy intake according to the years when the studies were conducted (r = -0.027, P = 0.785, Spearman correlation, Figure 3(a)). Also, no correlations were identified between prevalence of IBS and protein proportion (r = 0.172, P = 0.063, Pearson correlation, Figure 3(b)), carbohydrates proportion (r = -0.062, P = 0.505, Spearman correlation, Figure 3(c)), or fat proportion (r = 0.081, P = 0.384, Spearman correlation, Figure 3(d)).

### 3.4. Prevalence of IBS according to Diagnostic Criteria

**Figure 4:**
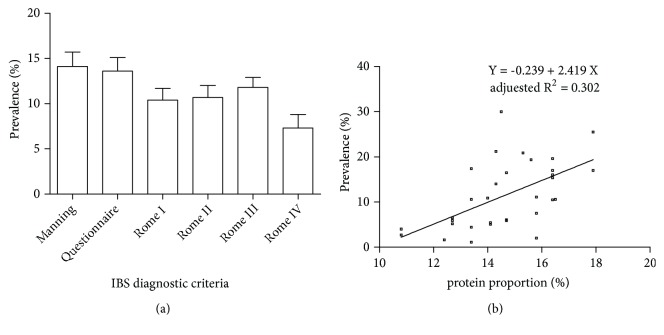
Prevalence of IBS according to diagnostic criteria. (a) The prevalence of irritable bowel syndrome (IBS) in Manning, questionnaire, Rome I, Rome II, Rome III, and Rome IV criteria (data were expressed as mean ± standard error). (b) The correlation between prevalence of IBS and protein proportion in Rome III criteria (Pearson correlation: r = 0.569, P ≤ 0.001; linear regression: R = 0.569, adjusted R^2^ = 0.302, F = 15.300, P ≤ 0.001, ANOVA).

In total, 22 studies used the Manning criteria, 20 used symptom questionnaire, 26 used the Rome I criteria, 44 used the Rome II criteria, 46 used the Rome III criteria, 4 used the Rome IV criteria, and 1 used unspecified Rome criteria. The prevalence of IBS in diagnostic criteria was shown in Figure 4(a). There was no significant difference in the prevalence of IBS according to diagnostic criteria (F = 1.184, P = 0.318, ANOVA).

We also explored the correlation between prevalence of IBS and dietary intake by different diagnostic criteria (Table 1). Overall, there was no correlation between prevalence of IBS of individual countries and energy intake, carbohydrates proportion, or fat proportion in the following five criteria. However, protein proportion was positively correlated with the prevalence of IBS in Rome III criteria (Pearson correlations: r = 0.569, P ≤ 0.001; linear regression: R = 0.569, adjusted R^2^ = 0.302, F = 15.300, P ≤ 0.001, ANOVA, Figure 4(b)).

### 3.5. Prevalence of IBS with Gender

The female prevalence of IBS was 13.8% (95% CI 12.2–15.4%) in the world, while the male prevalence was 9.4% (95% CI 8.2–10.7%).  Table 2 indicated no correlation between dietary intake and male or female prevalence.

## 4. Discussion

This review collected data from all available population-based cross-sectional surveys that reported the prevalence of IBS. Global prevalence of IBS was 11.7% which was similar to the pooled IBS prevalence in systematic reviews published in 2012 (11.2%) and 2014 (11.9%) [2, 15]. According to geographic location, the prevalence of IBS was 10.0%, 11.0%, 12.8%, 13.1%, 16.5%, and 17.7% in Asia, North America, Europe, Australia, Africa, and South America, respectively, which was also similar to the data shown in the review of 2012 [2]. According to study year, the prevalence of IBS was 8.1%, 10.8%, 12.2%, 12.0%, 11.3%, and 17.7% in 1970-1979, 1980-1989, 1990-1999, 2000-2009, and 2010-present, respectively, suggesting that there was no upward or downward trend in prevalence of IBS over the past five decades. The result was consistent with the former studies [2, 15].

In the past 20 years, a number of researches have investigated the role and possible mechanism of gender in prevalence of IBS [16–18]. It is now widely accepted that prevalence of IBS is higher in women, also supported by our results. It may be due to the fact that women are more vulnerable to be influenced by psychosocial factors, for example, stress of daily life [18, 19].

Previous studies suggested that the prevalence of IBS in a certain population would vary by different diagnostic criteria [16, 20]. However, the prevalence of IBS presented no difference in different diagnostic criteria in our study.

Due to the lack of standardized age bands, it was difficult to pool all existing data about age distribution of subjects with IBS together in the previous studies. In 2012, a review suggested that prevalence of IBS appeared to decrease modestly with increasing age [2]. While in the present study, the majority of IBS was 30–50 years old (47.3%), and the two other groups who were younger than 30 years and older than 50 years old occupied almost quarter proportion in separation, which was in keeping with the results in the study of 2014 [15]. Accordant to the studies of 2012 and 2014, our study showed that a quite uniform bowel habit distribution by IBS subtypes worldwide [2, 15].

In this study, we found that there was no correlation between total prevalence of IBS of individual countries and their national energy intake, protein proportion, carbohydrates proportion, or fat proportion according to the years when the studies were conducted. The similar correlations were presented in male or female prevalence of IBS with dietary intake, suggesting that dietary energy and macronutrients intake may not play a direct role in prevalence of IBS at national level. It appeared to be consistent with the result of dietary surveys between IBS patients and community controls that the intake of calories, carbohydrates, proteins, and fat by IBS patients does not differ from the background population [9, 21–23].

We further explored the correlation between prevalence of IBS and dietary intake according to diagnostic criteria. Similarly, irrelevant correlations were obtained except that protein proportions were positively correlated with the prevalence of IBS in Rome III criteria. It seemed that IBS diagnostic criteria may have a bias in the correlation between prevalence of IBS and dietary intake.

The dietary protein that has attracted the most attention in IBS is gluten, consisting of two proteins (gliadin and glutenin), which is rich in wheat, barley, and rye and is widely accepted trigger of celiac disease [24]. Some IBS patients report worsened symptoms after ingestion of food containing gluten but with negative tests for celiac disease and wheat allergy. These patients are considered with nonceliac gluten sensitivity (NCGS) [25]. Although there is no explicit mechanism for NCGS, some potential mechanisms have been reported in recent years. One is associated with activation of innate immune. IBS patients with NCGS present with low-grade inflammation in intestinal mucosal biopsies, which was characterized by the infiltration of mast cell and increased expression of Toll like receptor 2 [26, 27]. Also, digestion of gluten has been demonstrated to increase the production of inflammatory cytokines in dendritic cells and monocytes systemically in plasma [24, 28]. Moreover, recent studies have shown an increased level of interferon *γ*, the representative cytokine of T helper 1 cell, in the intestinal biopsy specimen of NCGS patients, suggesting the participation of adaptive immune response in the pathogenesis [29]. Another proposed mechanism is altered intestinal permeability. IBS-D patients with a gluten diet have presented with a decreased expression of tight-junction protein and increased intestinal permeability compared with those in a gluten-free diet [30, 31]. And these effects are more notable in the subgroup of HLA-DQ2 or HLA-DQ8 positive NCGS patients with IBS-D [30, 31]. The next potential mechanism for NCGS is the opioid hypothesis. The hydrolysates of gluten are found to have opioid activity, so they may contribute to IBS related symptoms including abdominal pain, constipation, and abdominal distension [32].

Apart from gluten, there are other involved dietary proteins in IBS. Wheat-germ lectin and *α*-amylase/trypsin inhibitors have been exhibited to trigger innate immune response through mediation of Toll like receptor 4 [33, 34]. And lectin also show ability in impairment of intestinal permeability [33]. In addition, proteins from yeast and soy may also have an effect in IBS, since that a great proportion of IBS patients present with immune globulin E mediated allergy of these food [35].

Although there was no supporting evidence relating carbohydrates intake with IBS prevalence in the present study, a diet with low fermentable oligo-, di-, and monosaccharide and polyols has been an alternative choice for IBS patients with a growing body of evidence [36–38]. These carbohydrates are poorly digested or absorbed in the small intestine and can enter the colon, where they increase luminal osmotic pressure and induce gas production through fermentation of colonic bacteria, which can result in abdominal distension and pain [39–43]. Moreover, recent studies show that the byproducts of the interaction between FODMAPs and gut microbiota have an action on intestinal stem cells, resulting in an aberrant differentiation into endocrine cells and then leading to the development of visceral hypersensitivity, dysmotility, and abnormal intestinal secretion, all being features observed in IBS patients [44, 45].

This is the first study concerning the relation between prevalence of IBS and dietary intake at national level. We choose energy and three macronutrients intakes as major observation objects, and further studies focused on more detailed nutrients might be carried out to find more evidence on the pathogenesis of IBS.

## 5. Conclusion

Our findings demonstrate that dietary energy and macronutrients intake do not play a direct role in prevalence of IBS. However, IBS diagnostic criteria seem to have a bias on the correlation between prevalence of IBS and dietary intake. Further studies are needed to confirm the correlation between prevalence of IBS and specific dietary intake.

## Figures and Tables

**Table 1:** Correlation between prevalence of IBS and dietary factors according to diagnostic criteria.

Diagnostic criteria	Correlation coefficients	P value	Statistical methods
Manning			
Energy intake/kcal	0.029	0.936	Spearman
Protein%	0.093	0.715	Spearman
Carbohydrates%	-0.349	0.156	Spearman
Fat%	0.252	0.313	Pearson

Questionnaire			
Energy intake/kcal	0.029	0.936	Pearson
Protein%	-0.507	0.065	Spearman
Carbohydrates%	0.535	0.060	Spearman
Fat%	0.104	0.724	Spearman

Rome I			
Energy intake/kcal	-0.362	0.128	Spearman
Protein%	0.255	0.253	Spearman
Carbohydrates%	0.104	0.644	Spearman
Fat%	-0.045	0.843	Spearman

Rome II			
Energy intake/kcal	-0.167	0.414	Spearman
Protein%	0.141	0.483	Spearman
Carbohydrates%	0.076	0.706	Spearman
Fat%	0.033	0.869	Spearman

Rome III			
Energy intake/kcal	0.063	0.742	Pearson
Protein%	0.569*∗*	≤0.001	Pearson
Carbohydrates%	-0.313	0.710	Spearman
Fat%	0.114	0.522	Spearman

IBS: irritable bowel syndrome; *∗* P < 0.05 (2-tailed); %: percent contribution to energy intake.

**Table 2:** Correlation between male or female prevalence of IBS and dietary factors.

Gender	Correlation coefficients	P value	Statistical methods
Male			
Energy intake/kcal	-0.203	0.105	Spearman
Protein%	0.088	0.950	Pearson
Carbohydrates%	0.089	0.462	Spearman
Fat%	-0.101	0.403	Spearman

Female			
Energy intake/kcal	0.013	0.920	Spearman
Protein%	0.061	0.613	Pearson
Carbohydrates%	-0.087	0.469	Spearman
Fat%	0.104	0.390	Spearman

IBS: irritable bowel syndrome; %: percent contribution to energy intake.

## Supplementary Materials

Supplementary Materials Table S1. Characteristics of included studies.
