# Supplementary material for: The Role of Dietary Energy and Macronutrients Intake in Prevalence of Irritable Bowel Syndromes
Source: Biomed Res Int. 2019 May 16;2019:8967306. doi: 10.1155/2019/8967306 (PMC6541956; doi:10.1155/2019/8967306)
Supplement: Supplementary Materials — Table S1. Characteristics of included studies. [file 8967306.f1.pdf]

Table S1. Characteristics of included studies

First author	Publish year	Study year	Country	Sample size	IBS diagnostic criteria	Prevalence (%)	Male prevalence (%)	Female prevalence (%)	Year of nutrition survey	Energy intake (kcal)	protein proportion (%)	Carbohydrates proportion (%)	Fat proportion (%)
Agreus	1995	1988	Sweden	1156	Questionnaire	12.5	N/A	N/A	1981-1983	2975	13.8	50.8	36.1
Drossma	1993	1990	USA	5430	Rome I	11.2	7.7	14.5	1988-1994	N/A	15.0	49.8	33.5
Jones	1992	1987	UK	1620	Rome I	21.6	18.7	24.3	1981-1983	3155	11.6	51.2	37.3
Kennedy	1998	1994	UK	3169	Manning	17.2	10.5	22.9	1991-1993	3263	12.0	50.6	37.7
Aggarwal	2006	2003-2004	UK	2299	Rome II	9.2	N/A	N/A	2001-2003	3444	12.6	53.0	34.0
Agreus	2000	1995	Sweden	1139	Manning	21.9	N/A	N/A	1991-1993	3095	13.6	51.5	36.1
					Rome I	16.9	N/A	N/A	1991-1993	3095	13.6	51.5	36.1
					Questionnaire	15.1	N/A	N/A	1991-1993	3095	13.6	51.5	36.1
Amra	2006	2002-2003	Iran	4762	Rome II	7.1	N/A	N/A	2005	2257	13.4	58.4	31.5
Andrews	2005	2001-2002	USA	25986	Rome II	6.6	N/A	N/A	1999-2002	N/A	15.3	50.7	33.0
Aro	2004	1998	Sweden	2122	Questionnaire	25.6	N/A	N/A	1991-1993	3095	13.6	51.5	36.1
Baretic	2002	1997	Croatia	500	Rome I	28.2	19.8	36.8	2012-2013	1822	17.6	45.5	36.4
Basaranoglu	2008	2003	Turkey	707	Rome II	8.6	N/A	N/A	N/A	N/A	N/A	N/A	N/A
Boekema	2001	1996	Netherlands	428	Manning	5.8	3.2	7.9	1991-1993	3285	13.1	52.0	35.7
Bommelaer	2002	1999	France	11131	Rome I	4	2.5	5.3	1991-1993	3537	13.5	44.8	40.8
Bommelaer	2004	2000	France	8221	Manning	2.5	1.7	3.1	2001-2003	3643	14.1	45.6	41.0
					Rome I	2.1	1.4	2.8	2001-2003	3643	14.1	45.6	41.0
					Rome II	1.1	0.9	1.3	2001-2003	3643	14.1	45.6	41.0
Boyce	2000	1995	Australia	2910	Manning	13.6	9.7	17.2	1991-1993	3516	12.7	47.0	39.5
					Rome I	4.4	2.2	6.4	1991-1993	3516	12.7	47.0	39.5
					Rome II	6.9	4.5	9.2	1991-1993	3516	12.7	47.0	39.5
Caballero	1999	1995	Spain	264	Rome I	13.6	10.7	16.2	1991-1993	3279	13.7	47.0	39.1
Campos	2001	1996	Peru	300	Manning	26	26.5	25.6	N/A	N/A	N/A	N/A	N/A

Celebi	2004	1999	Turkey	1766	Rome II	6.3	5	7.4	N/A	N/A	N/A	N/A	N/A
Chen	2000	1995	Singapore	271	Manning	2.6	N/A	N/A	1993	1872	14.5	55.2	30.3
Cheung	2007	1996	Hong Kong	1649	Rome I	4.1	N/A	N/A	N/A	N/A	N/A	N/A	N/A
Choo	2000	1995	South Korea	420	Manning	15.5	15	15.9	2012	2237	11.4	59.0	15.0
					Rome I	8.6	8	9.1	2012	2237	11.4	59.0	15.0
Corazziari	2008	2003	Italy	29139	Questionnaire	7.9	5.5	10.7	2001-2003	3670	12.9	49.7	37.7
Curioso	2002	1997	Peru	231	Manning	22.1	24.7	20.8	N/A	N/A	N/A	N/A	N/A
Danivat	1988	1983	Thailand	401	Questionnaire	5.7	N/A	N/A	2004-2005	1408	15.8	61.4	22.8
Dapoigny	2004	2001	France	15120	Rome II	4.7	3.7	5.7	2001-2003	3643	14.1	45.6	41.0
Drug	2000	1995	Romania	338	Rome I	14.5	8.4	17.7	N/A	N/A	N/A	N/A	N/A
Ebling	2011	2006	Croatia	703	Rome III	29.2	21.4	36.7	2012-2013	1822	17.6	45.5	36.4
Gomez	2009	2004	Colombia	558	Rome III	9.9	N/A	N/A	N/A	N/A	N/A	N/A	N/A
Gwee	2004	1998-2000	Singapore	2276	Manning	11	9.5	12.6	1998	2087	13.4	60.1	26.5
					Rome I	10.4	9	11.7	1998	2087	13.4	60.1	26.5
					Rome II	8.6	7.8	9.4	1998	2087	13.4	60.1	26.5
Han	2006	2001	South Korea	1066	Rome II	6.6	7.1	6	2012	2237	11.4	59.0	15.0
Heaton	1992	1987	UK	1896	Rome I	9.5	5	13	1981-1983	3155	11.6	51.2	37.3
Herschbach	1999	1994	Germany	2201	Rome I	2.1	1.7	2.5	1991-1993	3365	13.3	50.0	36.4
Hillila	2004	2001	Finland	3631	Manning	9.7	8.3	11.2	2001-2003	3153	14.6	52.4	33.0
					Rome I	5.6	5.1	6.1	2001-2003	3153	14.6	52.4	33.0
					Rome II	5.1	5.1	5.3	2001-2003	3153	14.6	52.4	33.0
Ho	1998	1993	Singapore	696	Manning	2.7	N/A	N/A	1993	1872	14.5	55.2	30.3
Holtmann	1994	1989	Germany	423	Rome I	11.8	8.4	15.7	1981-1983	3359	13.5	50.1	34.8
Hongo	2011	2006	Japan	11020	Questionnaire	14	N/A	N/A	2006	1905	14.8	56.3	24.9
Husain	2008	2003	Pakistan	880	Rome II	13.3	13.1	13.4	N/A	N/A	N/A	N/A	N/A
Icks	2002	1998	Germany	1265	Rome II	11.8	12.3	11.3	1991-1993	3365	13.3	50.0	36.4
Jafri	2007	2002-2005	Pakistan	1048	Rome II	45	N/A	N/A	N/A	N/A	N/A	N/A	N/A

Jeong	2008	2000-2001	South Korea	1417	Rome II	2.2	1.8	2.6	2012	2237	11.4	59.0	15.0
Karaman	2003	2001	Turkey	1000	Rome II	19.1	N/A	N/A	N/A	N/A	N/A	N/A	N/A
Khademolhosseini	2011	2004	Iran	1978	Rome II	10.9	7.6	12.7	2005	2257	13.4	58.4	31.5
Khoshkrood	2009	2006-2007	Iran	18180	Rome III	1.1	0.7	1.5	2005	2257	13.4	58.4	31.5
Kumano	2004	2000	Japan	4000	Rome II	6.2	4.5	7.8	2000	1948	16.0	54.6	26.5
Kwan	2002	2000	Hong Kong	1000	Rome II	6.6	N/A	N/A	N/A	N/A	N/A	N/A	N/A
Lau	2002	1997	Hong Kong	1298	Rome II	3.7	3.6	3.8	N/A	N/A	N/A	N/A	N/A
Lee	2009	2004	Hong Kong	2005	Rome III	5.4	4.6	6.2	N/A	N/A	N/A	N/A	N/A
Lee	2009	2005-2006	South Korea	1443	Rome II	9.5	N/A	N/A	2012	2237	11.4	59.0	15.0
Li	2003	2001	Canada	437	Rome II	25.2	16.5	31	1990-1992	3017	15.4	51.0	36.0
Lydiard	1994	1980-1984	USA	13537	Questionnaire	1.1	N/A	N/A	1988-1994	N/A	15.0	49.8	33.5
Masud	2001	1997	Bangladesh	2426	Rome I	8.5	5.8	10.7	1995-1996	1977	7.2	86.2	6.6
					Questionnaire	24.4	20.6	27.7	1995-1996	1977	7.2	86.2	6.6
Mearin	2001	2000	Spain	2000	Questionnaire	14.1	8.6	19.3	2001-2003	3405	14.3	45.9	40.0
Nicholl	2008	2003	UK	6094	Rome II	13.8	N/A	N/A	2001-2003	3444	12.6	53.0	34.0
Okeke	2009	2004	Nigeria	443	Rome II	31.6	35.2	28.3	N/A	N/A	N/A	N/A	N/A
Olafsdottir	2005	2000	Iceland	1336	Manning	30.9	25.1	35.7	N/A	N/A	N/A	N/A	N/A
Osterberg	2000	1995	Sweden	2707	Rome I	10.6	7.4	13.3	1991-1993	3095	13.6	51.5	36.1
Pan	2000	1996	China	2486	Manning	8.7	N/A	N/A	1993	2412	11.9	64.5	23.1
					Rome I	1.1	N/A	N/A	1993	2412	11.9	64.5	23.1
Papatheoridis	2005	2000	Greece	700	Questionnaire	21.4	14.3	27.8	2001-2003	3682	12.5	51.3	37.6
Perveen	2009	2004-2005	Bangladesh	1503	Rome II	7.7	6.8	8.6	1995-1996	1977	7.2	86.2	6.6
Rajendra	2004	1999	Malaysia	949	Rome II	15.6	6.8	8.6	2003	1606	14.0	59.0	27.0
Reshetnikov	2009	2004	Russia	1040	Rome I	19	12.2	25.2	2002-2005	2556	17.2	46.0	36.8
Saito	2000	1996	USA	643	Manning	13.7	11.4	15.7	1988-1994	N/A	15.0	49.8	33.5
					Rome I	7.6	6.2	8.9	1988-1994	N/A	15.0	49.8	33.5
Segal	1984	1979	South Africa	332	Questionnaire	8.1	N/A	N/A	2002	2038	14.4	60.5	25.1

Soares	2005	2000	Brazil	183	Rome II	16.9	10.3	20	2008-2009	1902	17.0	56.0	27.0
Sperber	2007	2002	Israel	981	Rome II	2.9	1.8	3.7	2002	1603	15.2	54.0	30.8
					Rome III	11.4	9.5	13	2002	1603	15.2	54.0	30.8
Talley	1992	1988	USA	835	Manning	16.8	N/A	N/A	1988-1994	N/A	15.0	49.8	33.5
					Rome I	6.9	N/A	N/A	1988-1994	N/A	15.0	49.8	33.5
Talley	1994	1989	USA	919	Manning	14.1	9.4	18.4	1988-1994	N/A	15.0	49.8	33.5
Talley	1995	1988-1993	USA	3022	Manning	17.7	N/A	N/A	1988-1994	N/A	15.0	49.8	33.5
Talley	1995	1990	Australia	99	Manning	17.2	N/A	N/A	1991-1993	3516	12.7	47.0	39.5
Talley	1997	1995	Australia	730	Manning	13.2	N/A	N/A	1991-1993	3516	12.7	47.0	39.5
					Rome I	12.3	6.9	17	1991-1993	3516	12.7	47.0	39.5
Thompson	2002	1998	Canada	1149	Rome I	13.5	8.5	18.2	1990-1992	3017	15.4	51.0	36.0
					Rome II	12.1	8.7	15.3	1990-1992	3017	15.4	51.0	36.0
Usai	2010	2005	Italy	1900	Rome II	7.2	5.9	13.7	2001-2003	3670	12.9	49.7	37.7
Walter	2010	2005	Sweden	226	Manning	15.5	N/A	N/A	2001-2003	3444	14.2	51.5	35.2
					Rome II	12.8	N/A	N/A	2001-2003	3444	14.2	51.5	35.2
Wei	2001	2000	China	2892	Rome II	5.6	4.7	6.6	2000	2394	12.1	62.0	25.9
Whitehead	1982	1977	USA	832	Questionnaire	8.1	N/A	N/A	1988-1994	N/A	15.0	49.8	33.5
Wilson	2004	1999	UK	4807	Rome II	8.3	6.6	14	1991-1993	3263	12.0	50.6	37.7
Xiong	2004	2002	China	4178	Manning	11.5	9.7	13	2000	2394	12.1	62.0	25.9
					Rome II	5.7	5	6.3	2000	2394	12.1	62.0	25.9
Yilmaz	2005	2003	Turkey	3000	Rome II	10.3	8	12.4	N/A	N/A	N/A	N/A	N/A
Zagari	2010	2000-2004	Italy	1033	Rome I	7.1	N/A	N/A	2001-2003	3670	12.9	49.7	37.7
Zhao	2010	2007-2008	China	16078	Rome II	4.6	4.1	5	2006	2344	12.0	58.0	30.0
Buscail	2017	2013	France	41682	Rome III	5.4	4.8	5.4	2006-2007	2273	14.1	57.0	28.9
Lee	2014	2009	Korea	1362	Rome II	12	N/A	N/A	2012	2237	11.4	59.0	15.0
Mansouri	2017	2009	Iran	1850	Rome II	21.6	15.9	27.1	2005	2257	13.4	58.4	31.5
Rasmussen	2015	2010	Denmark	47174	Rome III	10.5	7.9	12.8	2003-2008	2125	16.4	49.7	33.9

Satake	2015	2013	Japan	993	Rome III	6.1	5.5	6.5	2013	1887	14.7	55.3	25.9
Lopez	2012	2005	Mexico	500	Rome II	16	9.2	20.3	N/A	N/A	N/A	N/A	N/A
Arasteh	2018	2013	Iran	9163	Rome IV	11.6	7.6	15.1	2010	1956	13.4	52.8	33.8
Choung	2015	2009	USA	3202	Questionnaire	13.6	N/A	N/A	2007-2010	N/A	15.6	49.5	33.1
Siah	2016	2011	Singapore	297	Rome III	20.9	18.6	22.3	2010	2624	15.3	52.1	31.4
Krogsgaard	2016	2010	Denmark	5986	Rome III	15.4	N/A	N/A	2003-2008	2125	16.4	49.7	33.9
		2011		3750		17	N/A	N/A	2003-2008	2125	16.4	49.7	33.9
		2013		2781		19.6	N/A	N/A	2003-2008	2125	16.4	49.7	33.9
Lee	2013	2008	Malaysia	221	Rome III	10.9	10.6	11.1	2003	1606	14.0	59.0	27.0
Cabrera	2016	2016	Colombia	1207	Questionnaire	8.9	N/A	N/A	N/A	N/A	N/A	N/A	N/A
Guo	2014	2011	Taiwan	1096	Rome III	19.4	17.7	25.5	N/A	N/A	N/A	N/A	N/A
krogsgaard	2013	2010	Denmark	6112	Rome III	16	11.4	19.1	2003-2008	2125	16.4	49.7	33.9
Chirila	2017	2012	Romania	158	Rome	15.2	N/A	N/A	N/A	N/A	N/A	N/A	N/A
Lee	2016	2005-2006	Korea	3429	Rome II	10.9	10.5	11.3	2012	2237	11.4	59.0	15.0
Perveen	2014	2011	Bangladesh	3000	Rome III	12.9	12.5	13.3	1995-1996	1977	7.2	86.2	6.6
Husain	2012	2007	Romania	1682	Rome II	14.6	N/A	N/A	N/A	N/A	N/A	N/A	N/A
leelakusolvong	2012	2003-2004	Thailand	3120	Rome I	14.3	N/A	N/A	2004-2005	1408	15.8	61.4	22.8
Lankarani	2017	2012	Iran	1038	Rome III	17.4	15.4	19.1	2010	1956	13.4	52.8	33.8
Ghoshal	2016	2010-2012	India	2774	Rome III	2.7	2.9	2.5	2004	1834	10.8	78.6	10.6
Kumar	2016	2011	Bangladesh	3500	Rome III	7.2	N/A	N/A	1995-1996	1977	7.2	86.2	6.6
Koloski	2015	2011	Australia	767	Rome III	17	N/A	N/A	2011-2012	2304	17.9	44.9	30.5
Matsumoto	2013	2010	Japan	10000	Rome III	21.2	18.6	23.9	2010	1875	14.3	54.4	27.6
Choung	2013	2009-2010	USA	3515	Rome III	19.4	13.2	24.7	2007-2010	N/A	15.6	49.5	33.1
Cai	2015	2010-2011	China	2950	Rome III	1.6	N/A	N/A	2011	2192	12.4	54.6	33.0
Le Pluart	2015	2013	France	35447	Rome III	5.4	4.6	5.6	2006-2007	2273	14.1	57.0	28.9
Long	2017	2010	China	1999	Rome III	5.9	6	5.7	2009	2273	12.7	31.2	56.1
Ligaarden	2012	2001	Norway	4621	Rome II	8.4	6.2	10.1	2010-2011	2243	18.0	44.0	33.0

Miwa	2012	2010	Japan	15000	Rome III	14	N/A	N/A	2010	1875	14.3	54.4	27.6
Dantoft	2017	2011-2015	Denmark	7493	Questionnaire	11.6	7.6	14.9	2003-2008	2125	16.4	49.7	33.9
Yun	2012	2005-2006	Korea	3365	Rome II	1.1	N/A	N/A	2012	2237	11.4	59.0	15.0
Koloski	2012	2008	Australia	1002	Rome II	8.2	N/A	N/A	2001-2003	3444	12.6	53.0	34.0
Olafsdottir	2012	1996	Iceland	1340	Questionnaire	16.9	N/A	N/A	N/A	N/A	N/A	N/A	N/A
		2006		1180		17.2	N/A	N/A	N/A	N/A	N/A	N/A	N/A
		1988	USA	3805		8.3	N/A	N/A	1988-1994	N/A	15.0	49.8	33.5
		2003		2914		11.4	N/A	N/A	1999-2002	N/A	15.3	50.7	33.0
Makharia	2011	2008-2009	India	4767	Rome III	4	3.2	4.8	2004	1834	10.8	78.6	10.6
Almario	2016	2015	USA	71813	Rome III	2	N/A	N/A	2011-2014	N/A	15.8	48.6	33.6
Amieva	2014	2009	Mexico	2186	Rome III	7.6	4.7	9.5	N/A	N/A	N/A	N/A	N/A
Chang	2012	2005-2008	Taiwan	4275	Rome III	4.4	N/A	N/A	N/A	N/A	N/A	N/A	N/A
Chirila	2012	2007	Romania	193	Rome III	19.1	18.7	19.4	N/A	N/A	N/A	N/A	N/A
Gonzales	2012	2011	Peru	200	Rome III	15	11.8	16.9	N/A	N/A	N/A	N/A	N/A
Hayatbakhsh	2013	2010-2011	Iran	2259	Rome III	4.4	N/A	N/A	2010	1956	13.4	52.8	33.8
Iri	2017	2013-2014	Iran	430	Rome III	10.6	11.5	10	2010	1956	13.4	52.8	33.8
Kanazawa	2016	2011	Japan	30000	Rome III	16.5	15.5	17.4	2011	1846	14.7	55.6	25.8
Koloski	2017	2012	Australia	3576	Rome III	25.5	N/A	N/A	2011-2012	2304	17.9	44.9	30.5
Krogsgaard	2014	2013	Denmark	2796	Rome III	19.6	N/A	N/A	2003-2008	2125	16.4	49.7	33.9
Liang	2015	2010	China	1214	Rome III	5.2	N/A	N/A	2009	2273	12.7	31.2	56.1
Liu	2014	2009	China	2773	Rome III	6.5	N/A	N/A	2009	2273	12.7	31.2	56.1
Palsson	2016	2011	USA	1949	Rome III	11.1	N/A	N/A	2011-2014	N/A	15.8	48.6	33.6
					Rome IV	6.1	N/A	N/A	2011-2014	N/A	15.8	48.6	33.6
			UK	1994	Rome III	10.6	N/A	N/A	2008-2012	1861	16.5	45.7	32.9
					Rome IV	5.5	N/A	N/A	2008-2012	1861	16.5	45.7	32.9
			Canada	1988	Rome III	11.7	N/A	N/A	1990-1992	3017	15.4	51.0	36.0
					Rome IV	5.8	N/A	N/A	1990-1992	3017	15.4	51.0	36.0

Palsson	2014	2013	USA	1216	Rome III	7.5	6.2	8.7	2011-2014	N/A	15.8	48.6	33.6
Qumseya	2013	2008	Palestine	1352	Rome III	30	N/A	N/A	1999-2000	2014	14.5	54.2	30.7
Sugawara	2018	2013	Japan	1002	Rome III	5.9	5	6.5	2013	1887	14.7	55.3	25.9
Torres	2018	2013	France	36448	Rome III	5.1	4.4	5.4	2006-2007	2273	14.1	57.0	28.9
Kjellstrom	2014	2000-2006	Sweden	2293	Questionnaire	26.2	18	33	2001-2003	3444	14.2	51.5	35.2
